# Effect of Basalt Powder on Hydration, Rheology, and Strength Development of Cement Paste

**DOI:** 10.3390/ma15238632

**Published:** 2022-12-03

**Authors:** Jiaming Li, Dehao Che, Zhihao Liu, Lan Yu, Xiaowei Ouyang

**Affiliations:** Research Center for Wind Engineering and Engineering Vibration, Guangzhou University, Guangzhou 510006, China

**Keywords:** surface properties, CSH formation, inter-particle force, crack propagation, interface strength, inert filler

## Abstract

Basalt materials (e.g., basalt powder, aggregate, and fiber) are commonly used in cement-based materials. To understand the mechanism of the influence of basalt on the properties of cement-based materials (i.e., fluidity, hydration, and strength), zeta potential tests with different Ca2^+^ concentrations were carried out using basalt powder (BP). It is found that BP has a weaker absorption for Ca2^+^ compared to cement and quartz particles, which is directly related to its surface chemical properties. This weak absorption has a significant influence on the rheology and early-age hydration of cement paste. Moreover, the morphology of hydrate on the surface of the material observed by scanning electron microscope (SEM) also shows that the growth of CSH on the surface of BP particles is smaller than that of cement particles, indicating that BP delays the formation of CSH. Rheological tests showed that the reduction of BP’s adsorption of calcium ions weakened the electrostatic repulsion between particles, which led to the reduction of rheological properties. The influence of BP on the strength of cement paste was studied through crack characterization and fracture observation. The results show that the interfacial strength between BP and hydration products is very weak and does not increase with the hydration process, and the chemical reaction of BP is not obvious. In addition, the substitution of BP for cement leads to a dilution effect. These factors cause the strength of cement paste to decrease.

## 1. Introduction

Cement production is a process with high energy consumption that generates 0.55~0.94 tons of CO_2_ emissions per ton [1]. It takes possession of 7% of the world’s total carbon dioxide emissions [2]. It is urgent to find alternatives to cement to reduce carbon dioxide emissions. Solid wastes such as fly ash, polishing slag, and marble dust are widely used as cement replacements [3,4,5]. Basalt powder (BP) is also used to partially replace cement to prepare concrete [6,7]. Basalt, mainly composed of oceanic crust, belongs to basic volcanic rock. It is also commonly used in cement-based materials as an aggregate and fiber due to its high strength, electrical insulation, corrosion resistance, and high-temperature resistance [8,9,10]. The researches on basalt materials in cement-based materials focus primarily on macro properties [11,12], lacking a deep explanation of the influence of its surface chemical properties on cement-based materials. To study the influence of basalt on the properties of cement-based materials (i.e., fluidity, hydration, and strength) at the micro and nanoscale, BP is used. In fact, BP is produced in large quantities around the world as a by-product, causing serious environmental pollution [7,13]. The results of this study will contribute to gaining a deeper understanding of the influence of basalt materials on the properties of cement-based materials and provide theoretical support for better use of BP in cement-based materials to reduce carbon emissions and environmental pollution.

BP is believed to be a mineral with a large specific surface area and high SiO_2_ and Al2O3 contents and can be used as supplementary cementing material [14]. BP is used as a micro-filler to make the aggregate surrounded by cement paste dense and thus strengthen the interface transition zone [15,16]. It was also believed that chemical reactions might occur at the surface of BP in the pore solution of cement paste, resulting in the nucleation of the CSH on its surface and thus strengthening the interface between basalt and matrix. Tasong et al. [17] studied the interaction between BP and ions in a cement solution. BP absorbed a large amount of hydroxide, sulfate, calcium, and potassium ions, and dissociated a large amount of silicate and alumina ions. This indicates that there is a distinct chemical interaction on its surface. Liu et al. [18] found that BP has a retardation effect on cement hydration. Li et al. [19] used 15% BP to replace cement to prepare UHPC with different curing conditions. The results showed that the strength of basalt concrete is 10.3% lower than that of reference concrete at ambient temperature, while that of basalt concrete is 10.8% and 7.3% higher than that of reference concrete under steam curing and autoclave curing, respectively. It is considered that BP has low reactivity at ambient temperature but becomes active at high temperatures. Saraya [20] also found that the pozzolanic reaction of BP was weak before 28d. Youness et al. [21] found that adding BP reduces the fluidity of cement-based materials. It was proposed that the decline in flowability of the mixed paste was caused by its sharp and rough surface. Its irregular shape makes the friction between particles increase. Moreover, it was believed that the large surface of BP absorbs a large amount of water, which reduces the lubrication between particles [16,18].

However, these studies do not take the effect of surface chemical properties into account. The research on the influence of BP’s surface chemical properties on cement-based materials is still limited, and its mechanisms have rarely been addressed. To further clarify the influence of BP, zeta potential was applied to reveal the surface chemical properties of BP. The morphology of hydration products on the BP surface at the early hydration stage (15 min, 1, 4, and 7 h) was observed by a scanning electron microscope (SEM). The effect of BP on hydration products was investigated by X-ray diffraction (XRD) and thermogravimetric analysis (TGA). The yield stress and plastic viscosity of cement paste blended with BP were tested to clarify the impact of BP on the rheological properties of cement paste. The crack propagation and fracture surface between BP and hydration products of hardened paste were analyzed to illustrate the influence of BP on the strength development of cement paste.

## 2. Materials and Methods

### 2.1. Materials and Mixture

**Figure 1:**
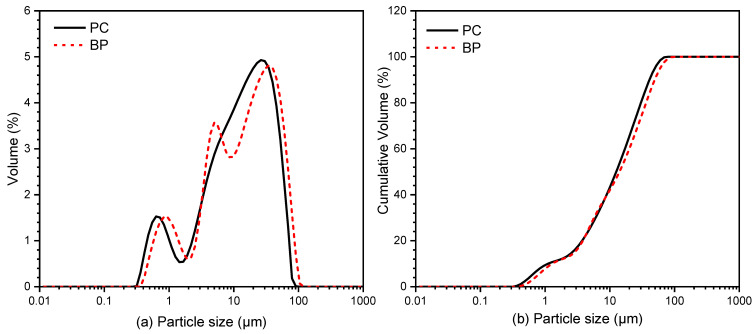
Particle size distribution (**a**) and cumulative particle size distribution (**b**) of OPC and BP.

**Figure 2:**
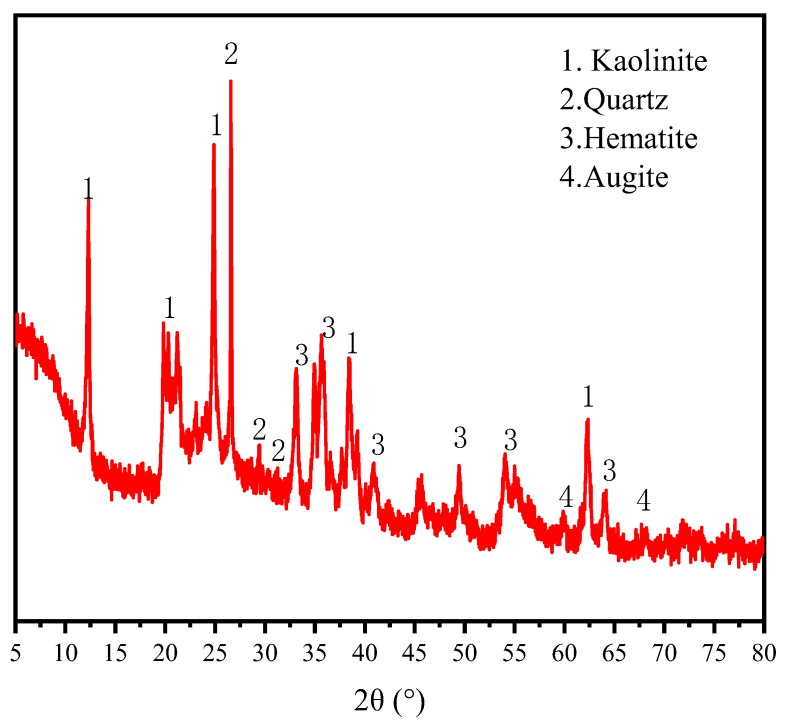
XRD pattern of BP. Phase identified: Kaolinite, Al_4_(OH)_8_(Si_4_O_10_), PDF No. 78-2109; Quartz, SiO_2_, PDF No. 79-1906; Hematite, Fe_2_O_3_, PDF No. 72-0469; Augite, FeO, PDF No. 46-1312.

**Figure 3:**
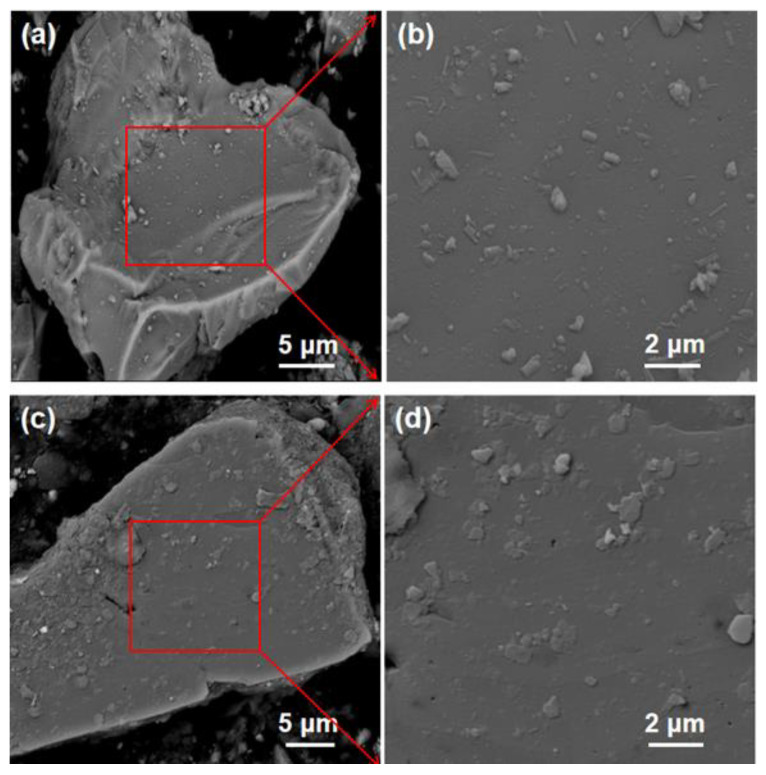
Morphology of OPC (**a**,**b**) and BP (**c**,**d**).

The cement model is P II 42.5 (OPC), its specific gravity is 3100 kg/m^3^, the initial setting time is more than 45 min, and the final setting time is less than 600 min. The BP came from the Yuxin building materials Co., Ltd. (Shijiazhuang, China). As OPC and quartz have similar surface charge properties [22,23], quartz powder (QP) purchased from Henan Birun Casting Materials Co., Ltd. (Zhengzhou, China) was used as a substitute for cement to do a zeta potential test to avoid the effect of cement dissolution on the test. Table 1 shows the chemical composition of the materials measured by a ZETIUM X-ray fluorescence spectrometer (XRF) (PANalytical Axios; RIGAKU ZSX Priums, PANalyticalB.V, Almelo, Netherlands). The sample shall be dried before the test, and the sample preparation is the tablet pressing method. The particle size was tested by a laser particle size analyzer (MS 2000, Malvern Instruments, Marvin City, Britain). The wet dispersion technology is adopted. The dispersant used for BP particle size analysis is pure water, and the dispersant used for cement samples is anhydrous ethanol. The method of mechanical agitation combined with ultrasonic dispersion was used to disperse the samples, and the results are depicted in Figure 1. It illustrates that BP is slightly larger than cement particles. The XRD diffraction of BP is shown in Figure 2. It is composed of kaolinite, quartz, hematite, and augite. The particle morphology of BP and OPC is shown in Figure 3. As can be seen, BP is a sharp-edged and split-like grain. The mixture ratio is shown in Table 2. During SEM analysis, it is not easy to find the BP in the BP paste when the proportion of BP substitution is low, and then it is not easy to understand the growth of its surface hydration products. However, in the rheological test, the rheological property of the paste is poor at a high BP substitution rate, and the test is difficult to carry out. BP paste prepared with BP instead of 20% cement is more in line with the test conditions. Therefore, the two groups of pastes prepared are OPC paste, and BP paste prepared by replacing 20% cement with BP, and the water-cement ratio is 0.4.

### 2.2. Zeta Potential Test

The formation of hydration products, inter-particle force in fresh cement paste, and the interface strength between the stone powder and hydrates are significantly affected by the surface charge properties of particles [24,25]. To compare the surface chemical properties of OPC and BP, zeta potential tests were applied. However, cement dissolves in the simulated solution, and its zeta potential is difficult to determine. It is mentioned in the literature [22,23] that the potential and morphology of quartz are similar to that of cement. Therefore, QP is used to replace cement for zeta potential testing to avoid the effect of cement dissolution on the test. Three series of synthetic interstitial solutions were prepared, and the interaction between ions and their surfaces was analyzed by zeta potential.

First, the zeta potential of particles was evaluated in NaOH solution with a pH ranging from 8 to 12. To understand the interaction of the particles with Ca^2+^ and SO_4_^2−^, the Ca(OH)_2_ solution and the mixed solution of Ca(OH)_2_ + K_2_SO_4_ were prepared. The chemical composition of the three model solutions is given in Table 3. Malvern Zetasizer Nano (ZS90, Malvern Instruments, Marvin City, Britain) was applied to measure the zeta potential. More detailed operation methods are shown in the literature [22].

### 2.3. Mechanical Performance

There are two groups of pastes in this compressive strength test, which are OPC paste and BP paste. Their mixtures are presented in Table 2. The samples were prepared in accordance with the standard procedure of ASTM C305 [26]. The prepared pastes were cast into cube molds with a size of 40 mm and then covered with plastic film. After 24 h, the samples were removed from the mold and cured in a standard curing chamber (humidity: 95 ± 5%, temperature: 20 ± 2 °C) till their respective age of testing. Three samples were taken and tested at each design curing age, and the mean value was used.

### 2.4. SEM Analysis

The Phenom ProX Electron microscope (Phenom, FEI, Eindhoven, Netherlands) was applied to evaluate the early hydration products, crack propagation, and fracture morphology; the accelerating voltage is 15 kV, and the backscattering mode was selected.

(1) Hydration product analysis. In this test, BP paste was placed in the curing room after preparation. About 1 g of paste was taken out in 15 min, 1, 4, and 7 h, respectively. These pastes were then immersed in absolute ethanol for hydration cessation. Afterward, these samples were filtered out and desiccated in a drying oven. The arid particles were used to analyze the formation of early hydration products on their surface.
(2) Fracture characteristics. The specimens at each curing age were loaded for 5 s under the compressive stress of 80% peak load. The loaded sample was cut into small test blocks with a side length of about 20 mm and immersed in absolute ethanol for more than 24 h to stop hydration. Then the test block was soaked in epoxy resin. The grinding and polishing steps of the sample were carried out after the epoxy resin had hardened. Afterward, the crack propagation of the sample was observed using SEM.
(3) Fracture surface analysis. The crushed pieces after the strength test were collected and halted hydration using absolute ethanol to observe the fracture morphology.

### 2.5. X-ray Diffraction Analysis (XRD)

After the strength test, the fragments were collected and ground with alcohol. The samples were then used to analyze the phase composition of the pastes at different curing ages. The instrument used for testing is the Bruker D max/RB diffractometer (Billerica, MA, USA) with the parameter of CuKα radiation (λ = 1.5418 Å), and the samples were scanned from 2θ = 5° to 2θ = 80° with a step size of 0.02°.

### 2.6. Thermogravimetric Analysis (TGA)

The TG/DTG data of the pure paste and the paste incorporating BP at the ages of 7, 28, and 96 d were obtained by using PerkinElmer TGA4000 (NETZSCH, Selb, Germany) The test was conducted using a heating rate of 10 °C/min from 30 °C to 800 °C in a nitrogen atmosphere at 1.5 bars.

### 2.7. Rheological Measurement

The rheological properties of pure paste and BP paste were tested by an RST Rheometer (Brookfield Ltd., Middleboro, MA, USA) accompanied by the CCT-40 slurry rotor. After being prepared, the paste was pre-sheared for the 60 s at a shear rate of 100 s^−1^, and then took a break for 60 s. Subsequently, the shear rate increased from 0 s^−1^ to 100 s^−1^ within the 30 s. After reaching the maximum shear rate, the shear rate decreased from 100 s^−1^ to 5 s^−1^ through 13 steps. To obtain reasonable results during the test of each step, 10 s were maintained in each step. The descending curve of the shear process was used as the final result. The Bingham-Plastic model was used to fit the shear stress-strain rate curve. Each paste was tested twice to obtain data on shear rate, shear stress, and viscosity, and the average value was used.

## 3. Results and Discussion

### 3.1. Surface Chemical Properties Analysis

#### 3.1.1. Effect of pH

**Figure 4:**
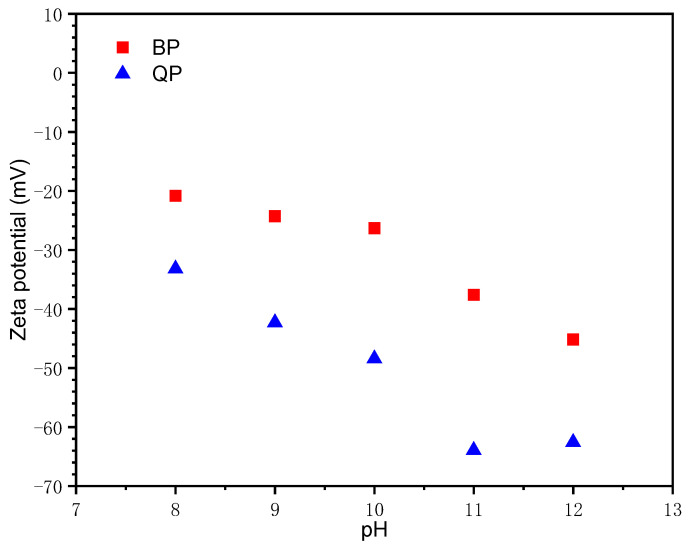
Zeta potential of BP and QP in NaOH solutions with pH from 8 to 12.

The zeta potential of BP and QP in NaOH solution is shown in Figure 4. As the pH increases from 8 to 12, the zeta potential of BP decreases from −20.83 mV to −45.17 mV, while that of QP decreases from −33.20 mV to −62.6 mV. In the solution, the silanol groups on the surface of QP undergo the following reactions [27,28,29].

≡SiOH + H_3_O^+^ ↔ ≡SiOH_2_^+^ + H_2_O (1)

≡SiOH + OH^−^ ↔ ≡SiO^−^ + H_2_O (2)

The formation of a positively charged surface is induced by the adsorption of protons on the SiOH surface (Equation (1)). In contrast, the SiOH surface was induced into a negatively charged surface under the reaction of Equation (2). One of the main components of BP is SiO_2_, so it may have the same reaction as quartz. However, the zeta potential of BP outstrips that of QP under the same alkaline conditions. This is mainly because QP dissociates more silanol groups under the action of OH^−^, resulting in lower potential. Nevertheless, BP with relatively less content of SiO_2_ has a small number of silanol groups. This surface chemical property is related to its weak adsorption of calcium ions, which will be further discussed in the next section.

#### 3.1.2. Effect of Ca^2+^ Concentration

**Figure 5:**
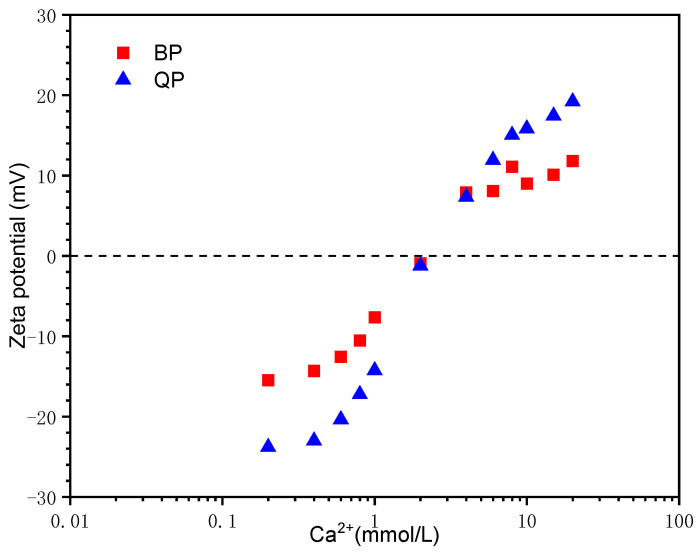
Zeta potential of BP and QP in Ca(OH)_2_ solutions.

Figure 5 shows the zeta potential of QP and BP in Ca(OH)_2_ solutions. QP is mainly composed of SiO_2_. These silicate-rich materials will ionize into SiO^−^ in an alkaline environment, resulting in the negative initial potential of particles, as mentioned. The initial potential of BP is also negative. As Ca^2+^ concentration increases, the potential of the two particles is rapidly raised. Their surface potential is balanced at the concentration of approximately 2 mmol/L Ca^2+^, which is the isoelectric point (IEP). This is because calcium ions adsorb on the particle’s surface, offsetting the negative potential. Furthermore, the increasing concentration of Ca(OH)_2_ makes more Ca^2+^ attach to the particle’s surface, and the potential is reversed from negative to positive. In addition, QP showed a higher potential after IEP, indicating that the adsorption capacity of QP for Ca^2+^ is stronger than that of BP.

#### 3.1.3. Effect of SO_4_^2−^ Concentration

**Figure 6:**
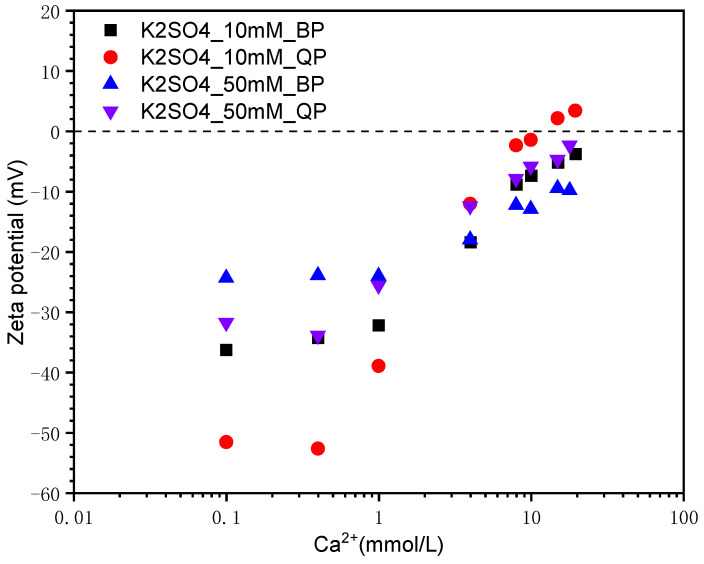
Zeta potential of BP and QP in the mixed solution of K_2_SO_4_ + Ca(OH)_2_.

The zeta potential of BP and QP in the mixed solution of K_2_SO_4_ + Ca(OH)_2_ is demonstrated in Figure 6. In the condition of the same Ca^2+^ concentration, the zeta potential of QP and BP in the Ca(OH)_2_ solution outstrips that in the K_2_SO_4_ + Ca(OH)_2_ solution, as shown in Figure 5 and Figure 6. It is inferred that these two particles have certain adsorption on SO_4_^2−^. In a mixed solution with K_2_SO_4_ of 10 mmol/L, the IEP of QP appears at the concentration of Ca^2+^ about 10 mmol/L. This illustrates that the affinity of QP for Ca^2+^ and SO_4_^2−^ is similar. However, the potential of BP has always been negative with the same K_2_SO_4_ concentration. It shows that the adsorption of Ca^2+^ on the BP’s surface is inferior to that of SO_4_^2−^. When the K_2_SO_4_ concentration is 50 mmol/L, the adsorption of Ca^2+^ on both QP and BP’s surface is insufficient to reverse the potential owing to the high concentration of SO_4_^2−^. Therefore, the zeta potential of both particles is negative. It is noted that the potential of QP is much higher than that of BP in solutions with a high Ca^2+^ concentration regardless concentration of SO_4_^2−^. It demonstrated that the interaction of Ca^2+^ with BP is much weaker than QP. This point will be further discussed in discussion.

### 3.2. Effect on the Hydration

#### 3.2.1. Morphology of Hydration Products

**Figure 7:**
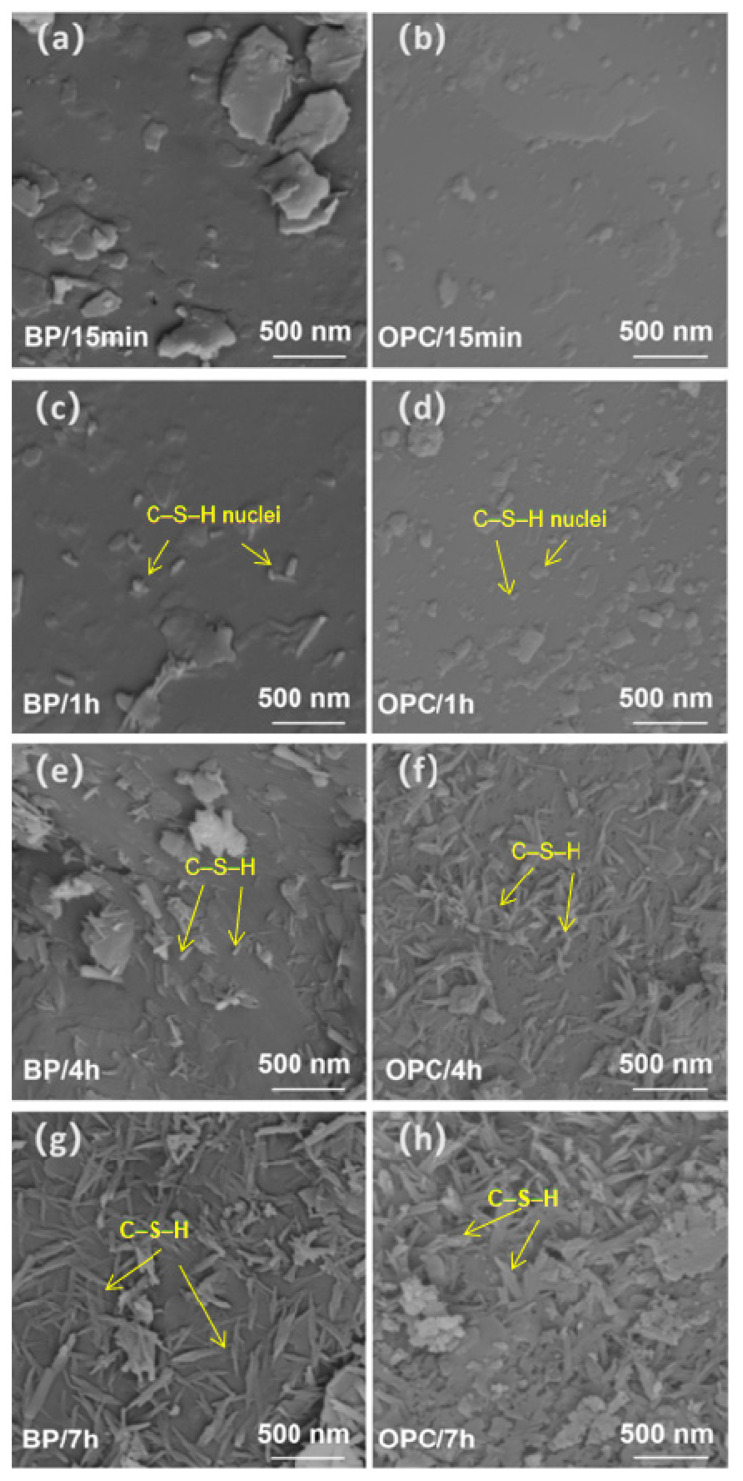
Hydrate morphology on the BP and OPC surface at 15 min (**a**,**b**), 1 h (**c**,**d**), 4 h (**e**,**f**) and 7 h (**g**,**h**).

Figure 7 exhibits the micrographs of hydration products on BP and OPC’s surface in the BP paste at different hydration times. Figure 7a–d shows the surface of BP and OPC after hydration for 15 min and 1 h. There are small dot-like particles on the surfaces, which may be the CSH nucleus [30]. After 4 h, the scattered CSH fibers can be seen at the BP and OPC’s surface, as displayed in Figure 7e,f. It is noticeable that the hydrates on the OPC surface are much more than that at the BP’s surface (Figure 7f). After hydration for 7 h, the size and length of hydration products increase. The OPC surface is almost wrapped by hydration products, while the BP’s surface was not completely covered by hydration products, as shown in Figure 7g,h. Ouyang et al. [25,31] found that cement particle has similar surface charge properties to quartz, and the adsorption of Ca^2+^ and the morphology of hydrates at their surface are also similar. As illustrated in Section 3.1, the adsorption of Ca^2+^ for QP is superior to BP. This indicates that the interaction of Ca^2+^ with OPC is stronger than that with BP. The SEM results here show that the amount and shape of hydration products on BP and OPC surfaces coincide with their interaction with Ca^2+^. It confirmed that the nucleation and growth of CSH are related to the ability of particles to adsorb Ca^2+^ [32]. This will be further discussed in discussion.

#### 3.2.2. XRD Analysis

**Figure 8:**
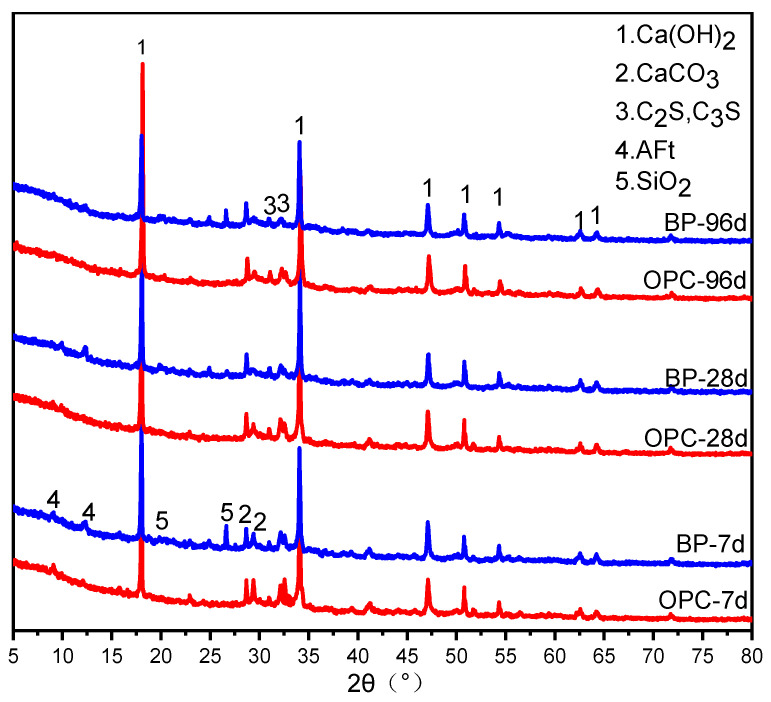
XRD patterns of OPC paste and BP paste at 7, 28, and 96 d. Phase identified: Ca(OH)_2_, PDF No. 84-1263; CaCO_3_, PDF No. 86-0174; C_3_S, C_2_S, PDF No. 73-0599; AFt, PDF No.41-1451; SiO_2_, PDF No.85-0798.

XRD data of OPC paste and BP paste at different curing ages is exhibited in Figure 8. At 7 and 28 d, both OPC paste and BP paste contain AFt, while CH existed at all ages. At 96 d, the crystal signals of C_3_S and C_2_S can still be observed in the pastes, which reveals that some cement particles are not fully hydrated. In addition, the diffraction peak of SiO_2_ was detected in BP paste, which is the main component of BP. The diffraction peak intensity of Ca(OH)_2_ in BP paste decreases slightly compared with the reference sample at their respective ages. However, even at the curing age of 96 d, there was very little change in phase in both pastes. It is difficult to speculate about the chemical reactivity of BP in the paste based on the XRD analysis.

#### 3.2.3. TGA Analysis

**Figure 9:**
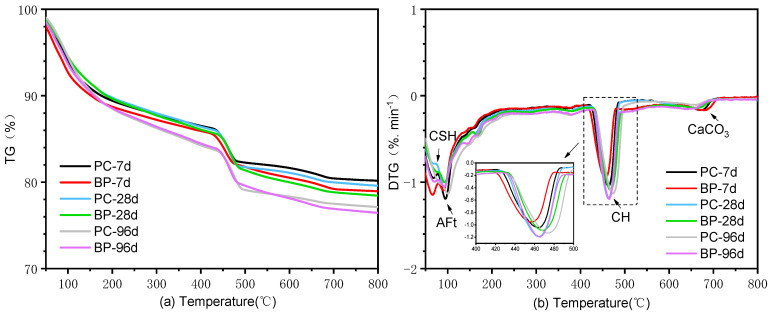
Weight loss of substance as a function of temperature (**a**) and weight loss rate of substance in unit time (**b**) of OPC paste and cement paste mixed BP hydration for 7, 28, and 96 d.

The thermal decomposition data of OPC paste and BP paste are shown in Figure 9. It is illustrated that there are three endothermic peaks. In the first endothermic peak, the mass loss is within 200 °C, matched with the release of physically bound water and the dehydration of Aft and CSH [33]. In the second endothermic peak, the mass loss is between 400~500 °C, corresponding to the decomposition of CH [34]. In the third peak, there is a small weight loss in the range of 650–750 °C, which is caused by the decomposition of CaCO_3_ [35]. In addition, it is well known that the consumption of CH in mixed cement paste is related to the degree of pozzolanic reaction [18,19]. However, the content of CH generated by the hydration of the two pastes is parallel and close at different curing ages. The difference in the content of CH in BP paste and OPC paste is mainly due to the decrease in cement content, which is the main source of liberated lime. Therefore, it is difficult to evaluate the reactivity of BP according to the consumption of CH. Based on TGA analysis, it can be concluded that the two groups of pastes have a similar weight loss trend regardless of curing age, and the results are consistent with the XRD analysis.

### 3.3. Effect on the Hydration

Yield stress and plastic viscosity are commonly applied to represent the rheological performance of paste. The yield stress determines the suspension stability of the paste and can be used to explain the results of a slump or collapse flow [36]. It is generally believed that fresh cement paste can be regarded as Bingham fluid [37]. For general viscoplastic materials, it must overcome the initial certain yield stress to initialize the flow. Nevertheless, after initialization, there is a linear relationship between the shear stress and shear rate, defined as plastic viscosity.

**Figure 10:**
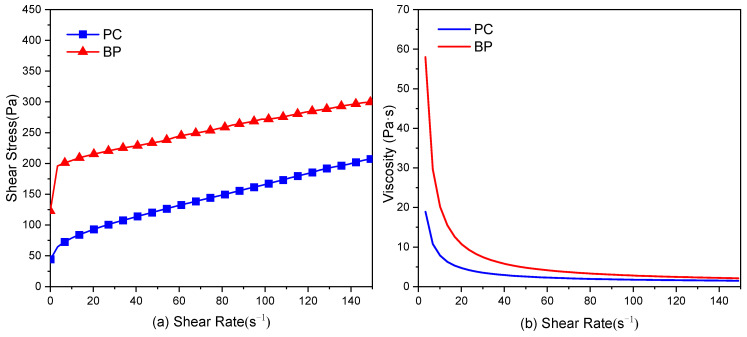
Rheological curve (**a**) and viscosity (**b**) of PC paste and BP paste.

The shear stress and viscosity vs. shear rate of OPC paste and BP paste are presented in Figure 10. As can be observed, the maximum shear stresses of OPC paste and BP paste are 207 and 299 Pa, respectively. In addition, the viscosity of the OPC paste is lower than that of the BP paste. These results exhibit that the fluidity of BP paste is inferior to that of OPC paste. Previous studies [24,38,39] have demonstrated that the interaction between mineral particles and cement particles affects the rheological performance of fresh cement paste, and this interaction is determined by the shape, size, and surface properties of the particles. Grains with diminutive particle sizes have a high bulk density. This leads to poor fluidity of the paste, while particles with bigger particle sizes are beneficial [40]. As can be seen from Figure 1, BP has large particle sizes but exhibits poor fluidity. This may be due to the particle’s morphology and surface chemical properties. Further explanation will be given in discussion.

### 3.4. Effect on the Strength Development

#### 3.4.1. Compressive Strength

**Figure 11:**
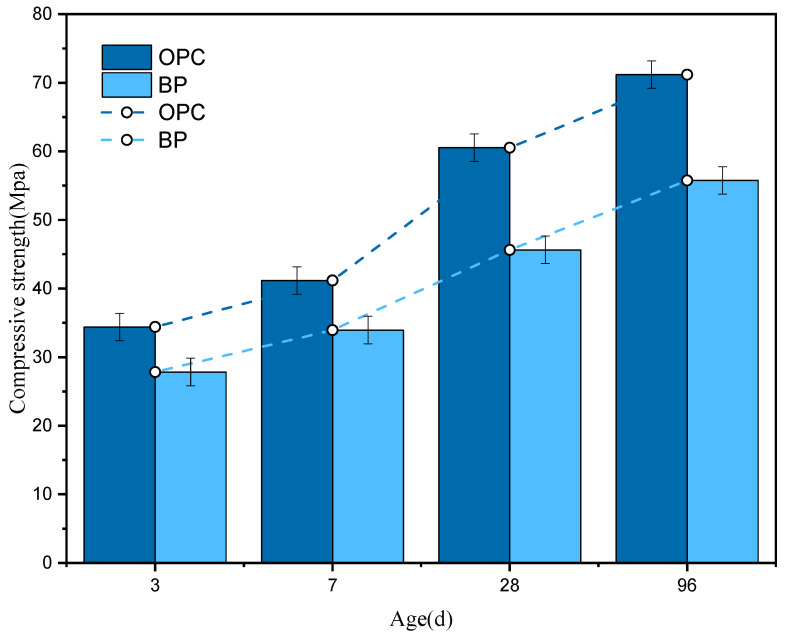
Compressive strength of the OPC pastes and BP pastes at different curing ages.

Figure 11 shows the compressive strength of OPC paste and BP paste at different ages. The strength of cement containing BP decreased by 19.1%, 17.5%, 24.6%, and 21.7% at 3, 7, 28, and 96 d, respectively. An exception occurred in the results at 7 d, in which the compressive strength is the closest to that of the control paste. This is due to the dilution effect [41,42]. After 7 d, the strength of the BP paste decreased by more than 20% compared with the reference paste. This may be related to the densification of the pore structure of the paste. When BP is used as a substitute for cement, the total cement content of the paste decreases. Correspondingly, the hydration products are also reduced. In addition, the lower strength of BP paste is also in connection to the weak reactivity of BP particles. Further analysis will be discussed in the next section.

#### 3.4.2. Crack Characterization

**Figure 12:**
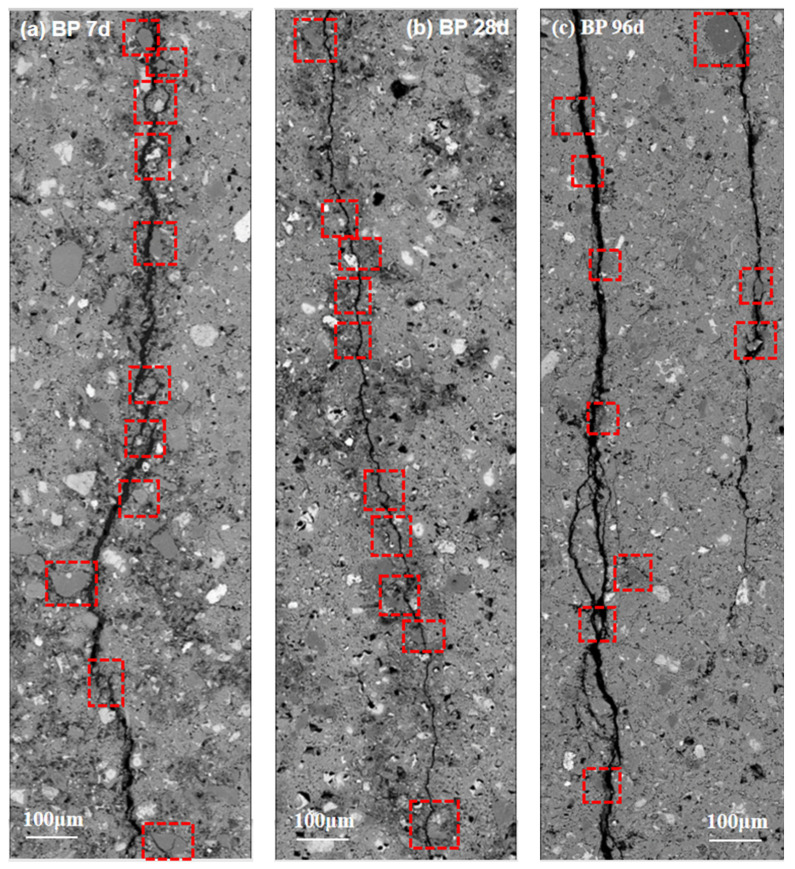
Cracks of hardened BP paste at 7 d (**a**), 28 d (**b**), 96 d (**c**) (The red box indicates the crack developed along the boundary of BP particle).

Previous research [43,44,45] has proved that crack propagation in cement paste is controlled by local stress distribution and weak interface. The interfacial strength can be characterized by the crack propagation between BP particles and hydration products. Figure 12 shows the common and representative crack propagation pattern in BP paste at various curing ages (7, 28, and 96 d). At the early stage of hydration, there is a rough interface at the crack, and the boundary between BP particles and paste is relatively clear, as shown in Figure 12a. However, the interface of the cracks becomes smooth, and the border between BP particles and paste becomes blurred, which is caused by the high degree of hydration and the dense hydration products filling the pore around it, as can be observed in Figure 12b,c. However, the cracks that extend along the boundary of BP particles can be observed regardless of curing time. This shows that the adhesion between BP particles and hydration products is always weak, even though the strength of the cement paste increased with the increasing curing age.

#### 3.4.3. Fracture Surface

**Figure 13:**
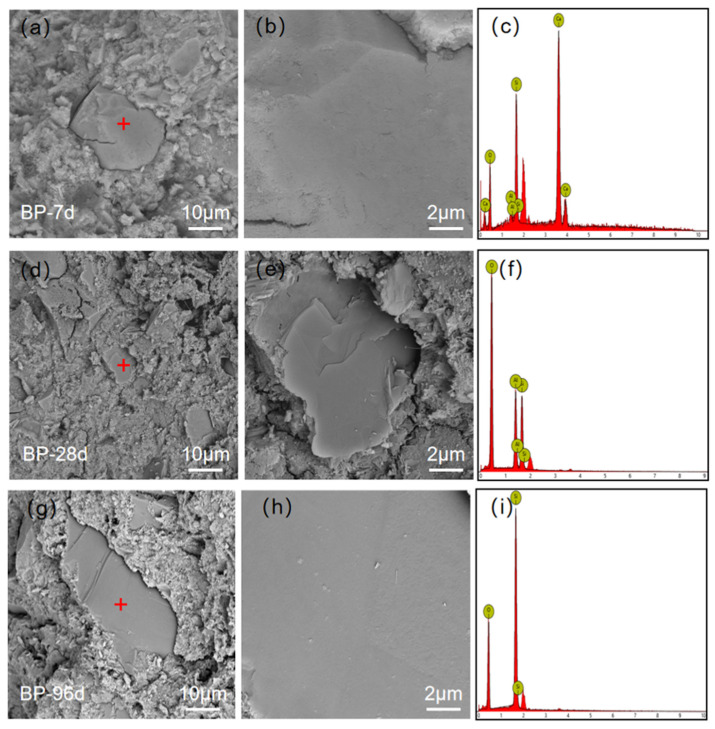
The fracture surface of the BP paste at 7 d (**a**–**c**), 28 d (**d**–**f**), 96 d (**g**–**i**).

To understand the weak interface, the fracture surface in BP paste was investigated, as shown in Figure 13. It can be observed that the surface of BP at the ages of 7 and 28 d is smooth, and almost no hydration products are attached to the particle surface. This is attributed to the weak interfaces between BP and hydration products. It is consistent with the cracks propagate observation. The fracture surface at 96 d is smooth, and the hydrates on the surface are not significant. The fracture propagation mode deduces the weak interface even at the curing age of 96 d. It is inferred that the reactivity of BP in cement paste is weak.

## 4. Discussion

Previous studies [32,46] have shown that Ca^2+^ absorbed on the surface of silica particles is controlled by electrostatic interaction. This force will drive Ca^2+^ and SO_4_^2−^ ions to be adsorbed on the particle’s surface. In the solution with higher Ca^2+^ concentration, BP exhibits lower zeta potential than QP. This may be due to its lower content of SiO_2_, which decomposes silanol groups in an alkaline environment, causing a decline in the probability of Ca^2+^ adsorption. Moreover, BP has complex components, and other components may have an effect, while QP has a high content of SiO_2_, which leads to its high potential. It is concluded from the zeta potential test that the interaction of BP with Ca^2+^ is weaker than that of OPC or QP. This interaction has a strong relation with the formation of hydration products, inter-particle force in fresh cement paste, and the interface strength between the stone powder and hydrates [22,24].

Studies have demonstrated that Ca^2+^ is a vital ion which not only plays a significant part in the morphological and structural characteristics of CSH [31,32] but is also in charge of the nucleation of CSH [47]. As described in Section 3.2, OPC and QP have similar adsorption capacities for Ca^2+^, and the growth of hydration products on their surfaces is also similar. QP or OPC has a stronger calcium ion adsorption capacity than BP, so the OPC surface presents denser hydration products on its surface. On the contrary, the amount of hydration products on the BP surface is much less due to its weak adsorption of calcium ions.

DLVO theory [48] shows an interaction force between charged surfaces in a liquid medium composed of van der Waals force and electrostatic repulsion caused by electric double layer (EDL) repulsion. The chemical properties of particle surfaces are important factors affecting the rheological performance of paste. Generally, particles with low zeta potential have lower electrostatic repulsion, which makes the particles difficult to disperse. Thus, the paste shows poor rheological properties. The addition of BP increases the shear stress of cement paste. Besides the particle size and morphology, the surface chemical properties play an important role in this increase. Its low adsorption capacity for Ca^2+^ results in a weak electrostatic repulsion between particles, thus promoting particle agglomeration in fresh cement paste.

As mentioned, crack propagation in cement paste is controlled by the weak interface. The crack in the cement paste blended with BP tended to propagate beside the boundary of BP particles in all test curing ages. It was demonstrated that although the strength of the paste increased significantly, the strength of the interface between BP and hydrates was not promoted. The crack feature showed that the interface strength between BP and hydrates is weak regardless of curing age. The smooth and clear fracture surface illustrated that the chemical activity of BP in cement paste is not obvious.

## 5. Conclusions

The surface chemical properties of BP were investigated. The influence of BP on the rheology of cement paste, the formation of hydrates, and the interface strength between BP and hydrates was studied. The conclusions are as follows:

(1) BP has a weak absorption of Ca^2+^. The surface chemical properties are directly responsible for this weak interaction.
(2) BP retards the formation of CSH on its surface and delays cement hydration. It is due to the weak absorption of Ca^2+^, which is crucial to the nucleation and development of CSH.
(3) The addition of BP increases the shear stress of cement paste. Besides the particle size and morphology, the surface chemical properties play an important role in this increase. Its low adsorption capacity for Ca^2+^ results in a weak electrostatic repulsion between particles, thus promoting particle agglomeration in fresh cement paste.
(4) The crack characteristics and fracture surface show that the interface strength between BP and hydrate is very weak regardless of the curing age. It also illustrates that BP has very low chemical activity. In addition, the substitution of BP for cement leads to a dilution effect. These factors cause the strength of cement paste to decrease.

## Figures and Tables

**Table 1:** Chemical compositions of materials (%).

Name	SiO2	CaO	Al_2_O_3_	MgO	K_2_O	Fe_2_O_3_	TiO_2_	NaO_2_	P_2_O_5_	Loss
OPC	19.15	63.05	4.62	2.06	0.70	3.80	0.40	0.35	0.55	1.7
BP	42.24	0.81	33.08	0.56	1.35	19.08	1.28	-	0.29	0.8
QP	97.5	-	1.49	-	0.75	0.25	-	-	-	-

**Table 2:** Mix proportions of paste.

Mixture	OPC (%)	Basalt Powder (%)	w/b
OPC paste	100	-	0.4
BP paste	80	20	0.4

**Table 3:** Mix proportions of paste the composition of the simulated solutions (mmol/L).

Solution 1		Solution 2		Solution 3	
NaOH	pH	Ca(OH)_2_	pH	Ca(OH)_2_	K_2_SO_4_
-	8	0.2	9.2	0.1	10
-	9	0.4	9.3	0.4	10
-	10	0.6	9.6	1	10
-	11	0.8	10.1	4	10
-	12	1	10.5	8	10
-	-	2	10.8	10	10
-	-	4	10.9	15	10
-	-	6	11	19.6	10
-	-	8	11.9	0.1	50
-	-	10	12	0.4	50
-	-	15	12.2	1	50
-	-	20	12.3	4	50
-	-	-	-	8	50
-	-	-	-	10	50
-	-	-	-	15	50
-	-	-	-	18	50

## Data Availability

Some or all data, models, or codes that support the findings of this study are available from the corresponding author upon reasonable request.
